# Differences in the metabolomic profile of the human palatine tonsil between pediatrics and adults

**DOI:** 10.1371/journal.pone.0288871

**Published:** 2023-07-31

**Authors:** Seokhwan Lee, Seonghye Kim, Sung-Dong Kim, Se-Joon Oh, Soo-Keun Kong, Hyun-Min Lee, Suhkmann Kim, Sung-Won Choi

**Affiliations:** 1 Department of Otorhinolaryngology, Inje University Haeundae Paik Hospital, Busan, Republic of Korea; 2 Department of Chemistry and Chemistry Institute for Functional Materials, Pusan National University, Busan, Republic of Korea; 3 Department of Otorhinolaryngology and Biomedical Research Institute, Pusan National University Hospital, Busan, Republic of Korea; 4 Department of Otorhinolaryngology and Biomedical Research Institute, Pusan National University Yangsan Hospital, Yangsan, Republic of Korea; University of California Riverside, UNITED STATES

## Abstract

Palatine tonsils (PT) are B cell-predominant lymphoid organs that provide primary immune responses to airborne and dietary pathogens. Numerous histopathological and immunological studies have been conducted on PT, yet no investigations have been conducted on its metabolic profile. We performed high-resolution magic angle spinning nuclear magnetic resonance spectroscopy-based metabolic profiling in 35 pediatric and 28 adult human palatine tonsillar tissue samples. A total of 36 metabolites were identified, and the levels of 10 metabolites were significantly different depending on age. Among them, partial correlation analysis shows that glucose levels increased with age, whereas glycine, phosphocholine, phosphoethanolamine, and ascorbate levels decreased with age. We confirmed the decrease in immunometabolic activity in adults through metabolomic analysis, which had been anticipated from previous histological and immunological studies on the PT. These results improve our understanding of metabolic changes in the PT with aging and serve as a basis for future tonsil-related metabolomic studies.

## Introduction

Palatine tonsils (PT) belong to mucosa-associated lymphoid tissue, is responsible for the primary immune response to airborne and alimentary pathogens introduced through the oral cavity or nasal cavity, and is mainly involved in the humoral immune response by antibody production [1–3].

The PT have four specialized tissue compartments (the reticular crypt epithelium, the extrafollicular area, the mantle zones of lymphoid follicles, and the follicular germinal centers) that contribute to immune function [1, 4]. Unlike the lymph nodes, the PT are not fully encapsulated and lack afferent lymph. Instead, the surface of the PT forms crypts to increase the contact surface area for pathogens, and numerous dendritic cells are present. Dendritic cells take up exogenous antigens and transport them to the extrafollicular T cell areas and B cell follicles [5, 6]. Antigen-presenting naive B cells are activated in the extrafollicular area. Some of these B cells become germinal center founder cells and undergo clonal expansion, somatic hypermutation, affinity maturation, and immunoglobulin class switching, finally differentiating into plasma cells or memory B cells [1, 6].

Another characteristic feature of PT is that morphological, histopathological, and immunological changes associated with age are distinct. The size of the PT is prominent during childhood but reduces in an age-dependent manner [7, 8]. Histopathologically, the parenchymal area and lymphoid follicle area decrease with age, and the fibrous connective tissue, collagen fiber, and elastic fiber areas increase with age [9]. Immunologically, the proportion of germinal center B cells decreases with age, and those of memory B cells increase with age. The Immunoglobulin (Ig) isoforms preferentially switch from IgM to IgA with age [10].

However, the age-related changes in the metabolic profile of the PT have not yet been studied.

For metabolic profiling, we used ^1^H nuclear magnetic resonance (NMR) spectroscopy. Typically, metabolic profiling using NMR spectroscopy requires a metabolite extraction procedure. The extraction procedure yields narrow and well-resolved ^1^H NMR spectra, however, it is crucial to consider the potential for erroneous metabolite concentrations due to the potential loss or degradation of metabolites [11, 12]. To mitigate this concern, we employed high-resolution magic angle spinning (HR-MAS) NMR, enabling direct measurements of intact tissue samples without metabolite extraction and with minimal sample preparation. In this study, we performed metabolic profiling on intact PT and compared the metabolic trends and differences between the pediatric and adult groups in PT. Furthermore, to confirm the validity of the results, the observed results were compared with results obtained from the extract solutions of the same PT samples.

## Materials and methods

### Participants and sample preparation

This study was approved by the ethics committee and the Institutional Review Board of Pusan National University Hospital and performed in accordance with the Declaration of Helsinki (H-2007-013-093). Informed consent was obtained from all participants and/or their legal guardians.

PT tissues were obtained from patients (N = 63, 36 male and 27 female, age range 3–70 years) who underwent palatine tonsillectomy for obstructive sleep apnea owing to chronic tonsillar hypertrophy. Patients without a history of acute tonsillitis for at least six months before surgery were included in the study. None of the participants had any diseases affecting their metabolism. Diet, body weight, or other medications were not assessed in this analysis. Tonsillectomy was performed under general anesthesia, and the operating time was not longer than an hour. Tonsil tissues were immersed in liquid nitrogen immediately after tonsillectomy and kept in a freezer at -80°C before NMR analysis.

### NMR measurement

For HR-MAS NMR, each sample was weighed to 20 mg and transferred to a 4-mm HR-MAS NMR tube (Agilent Technologies, Santa Clara, CA, USA), and 20 μL of phosphate buffer (pH 7.4) in deuterium oxide (D_2_O) containing 2 mM 3-(trimethylsilyl) propionic-2,2,3,3-d_4_ acid sodium salt (TSP-d_4_) was added. TSP-d_4_ was used as an internal standard for the chemical shift and quantification of metabolites. D_2_O and TSP-d_4_ were purchased from Sigma–Aldrich (St. Louis, MO, USA). HR-MAS NMR spectroscopy was used for intact tissue sample analysis without extraction. All NMR spectra were acquired using a 600 MHz Agilent spectrometer equipped with a 4-mm gHX NanoProbe (Agilent Technologies, Santa Clara, CA, USA). The spinning rate was set at 2070 Hz, and the CPMG (Carr–Purcell–Meiboom–Gill) pulse sequence was used to suppress high molecular mass compounds and water signals. The acquisition time was set at 3.0 s, 90-degree pulse (pw) was set at 8.7 μs, and the relaxation delay was set at 3 s. A total of 64 scans were acquired for each sample at a spectral width of 24,038.5 Hz.

For solution ^1^H NMR, extraction of PT tissue was performed based on the Bligh-Dyer extraction technique [13]. The extract solutions were analyzed using a 600 MHz Agilent spectrometer equipped with a 5 mm PFG One NMR Probe and CPMG pulse sequence. The acquisition time was set at 3.0 s, 90-degree pulse (pw) was set at 17.2 μs, and the relaxation delay was set at 1 s. A total of 64 scans were acquired for each sample at a spectral width of 24,038.5 Hz.

### Data analysis

All acquired spectras were manually phased and baseline-corrected. For multivariate statistical analysis, HR-MAS NMR spectra was binned from 0.9 to 8.6 ppm with a binning size of 0.04 ppm, after removing the residual water and sideband regions. The binned data were normalized to the total area and aligned using *icoshift* algorithm in MATLAB (MathWorks, Natick, MA, USA) [14]. For multivariate analyses, binned data were normalized with mean-centered scaling (Ctr) and imported into SIMCA P^+^ 12.0, software (Umetrics, Umeå, Sweden) to perform principal component analysis (PCA) and orthogonal partial least squares discriminant analysis (OPLS-DA).

For the identification and quantification of metabolites, Chenomx NMR Suite 7.1 Professional (Chenomx, Edmonton, AB, Canada) was used with a 600 MHz NMR library database and Human Metabolome Data Base (HMDB, www.hmdb.ca). Metabolites concentrations were normalized using probabilistic quotient normalization (PQN) method. To characterize metabolic changes in the tonsil during aging, data were grouped and analyzed according to two age groups (G1, group under 10 years old; G2, group over 20 years old). Two-way analysis of variance (ANOVA) was used to determine the interaction between age and gender in influencing the metabolite alterations. Partial correlation analysis was performed to measure the strength and direction of the linear relationship between age and metabolite concentration while controlling for the effect of gender. In all statistical tests, false discovery rate (FDR) was applied for multiple comparisons of individual metabolite levels. Statistical analyses were performed using the MetaboAnalyst software (version 5.0, www.metaboanalyst.ca).

## Results

### Metabolomics profile of tonsil tissue

**Fig 1:**
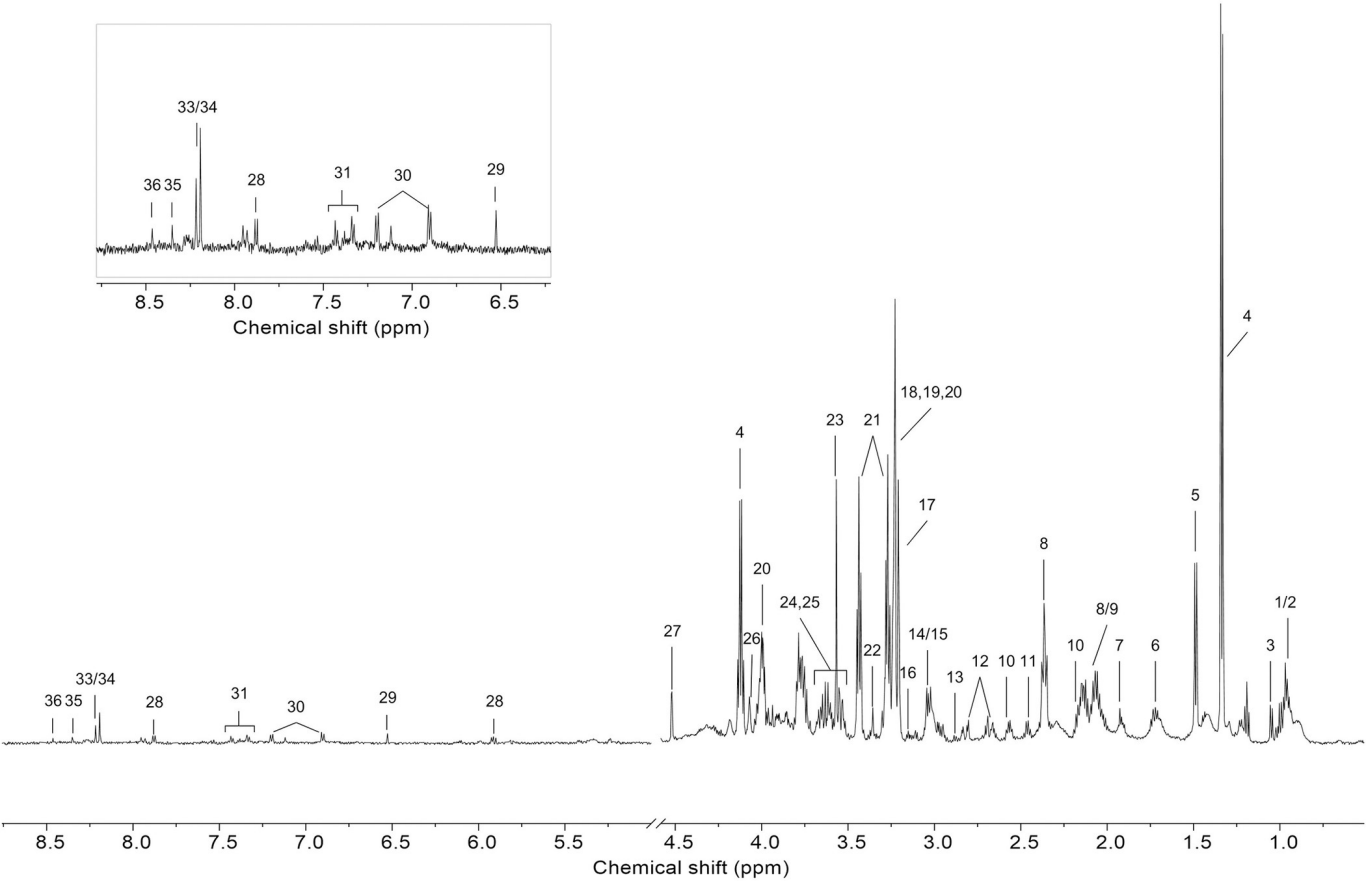
The representative 600 MHz HR-MAS ^1^H-NMR spectra of palatine tonsil tissue. Key: 1, isoleucine; 2, leucine; 3, valine; 4, lactate; 5, alanine; 6, lysine; 7, acetate; 8, glutamate; 9, methionine; 10, glutathione; 11, glutamine; 12, aspartate; 13, asparagine; 14, creatine; 15, creatine phosphate; 16, ethanolamine; 17, choline; 18, phosphocholine; 19, glycerophosphocholine; 20, phosphoethanolamine; 21, taurine; 22, proline; 23, glycine; 24, glycerol; 25, threonine; 26, myo-Inositol; 27, ascorbate; 28, uridine; 29, fumarate; 30, tyrosine; 31, phenylalanine; 33, oxypurinol; 34, niacinamide; 35, inosine; 36, formate.

Sixty-three individuals were recruited and divided into two groups: pediatric (under 10 years old; G1) and adult (over 18 years old; G2). A total of 35 participants were included in G1 (18 male and 17 female) and 28 in G2 (18 male and 10 female). The adult group contained fewer female than the pediatric group. Typical HR-MAS ^1^H-NMR spectra derived from tonsil tissues are shown in Fig 1. A total of 36 metabolites were identified, including carboxylic acids, amino acids, amines, carbohydrates, and other organic compounds (S1 Table).

### Multivariate analysis of NMR spectra

**Fig 2:**
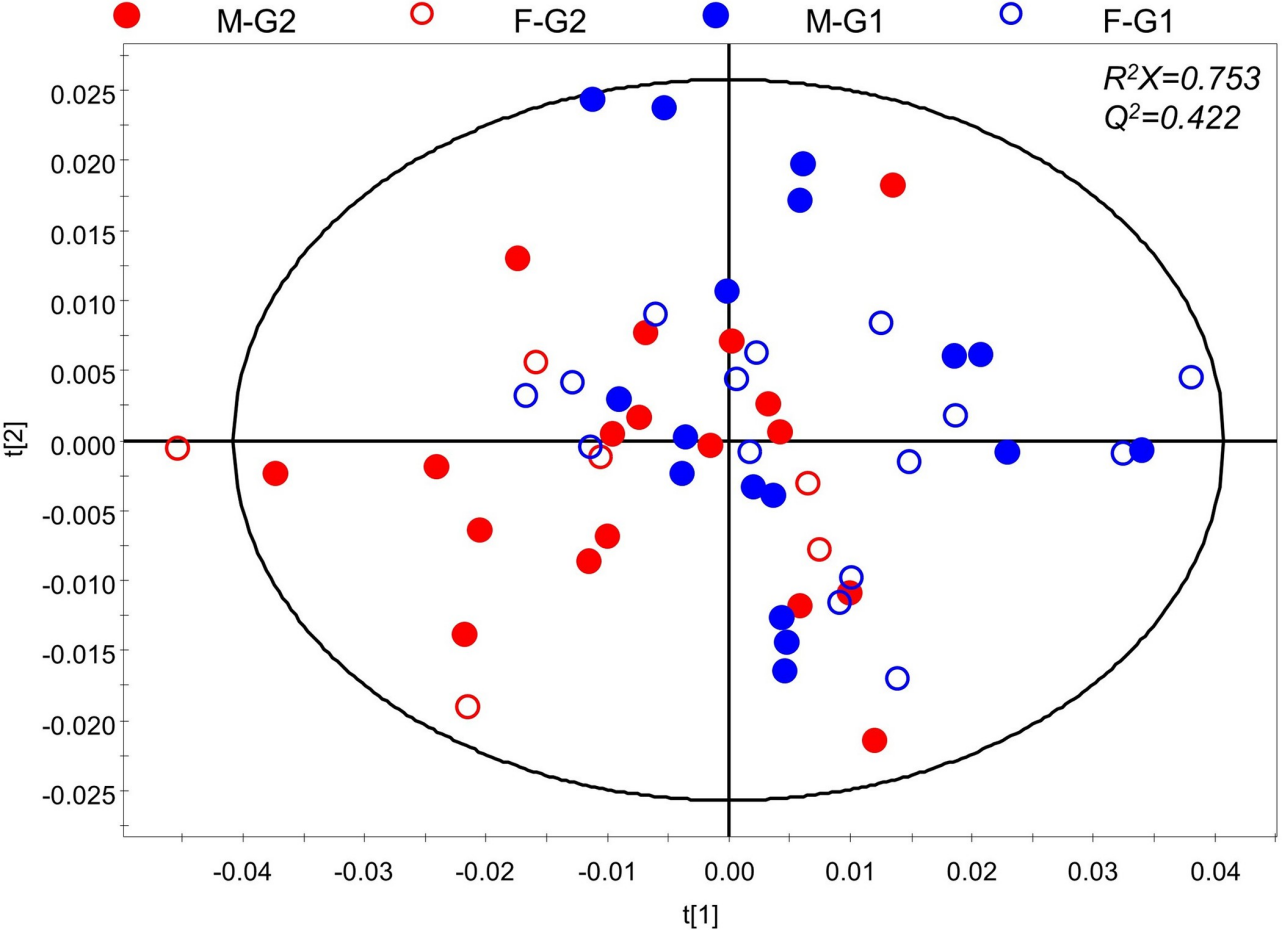
PCA score plot of tonsil tissue. The legend represents age group and gender. (●, male of G1; ○, female of G1; ●, male of G2; ○, female of G2).

NMR spectral data were processed using multivariate analysis. PCA is an unsupervised method that checks the distribution of the samples and outliers in the data. To detect outliers across the multidimensional space of PCA, the Hotelling’s T^2^ test was applied. Seven outliers exhibiting Hotelling’s T^2^ higher than the 95% confidence level were excluded and all the remaining subjects were in the defined confidence interval (S1 Fig). Fig 2 shows the PCA score plot of 56 samples after exclusion of outliers. The subsequent analyses were conducted with the remaining 56 samples, and their demographic characteristics are shown in Table 1.

**Table 1:** Demographic characteristics of the 56 participants.

	G1		G2		Total
	Male	Female	Male	Female	
**Individuals (n)**	17	15	18	6	56
**Age (years)**	5.52 ± 1.36	6.07 ± 1.74	34.22 ± 9.41	33.50 ± 7.76	17.89 ± 15.23
	5.78 ± 1.57		34.04 ± 9.10		

**Fig 3:**
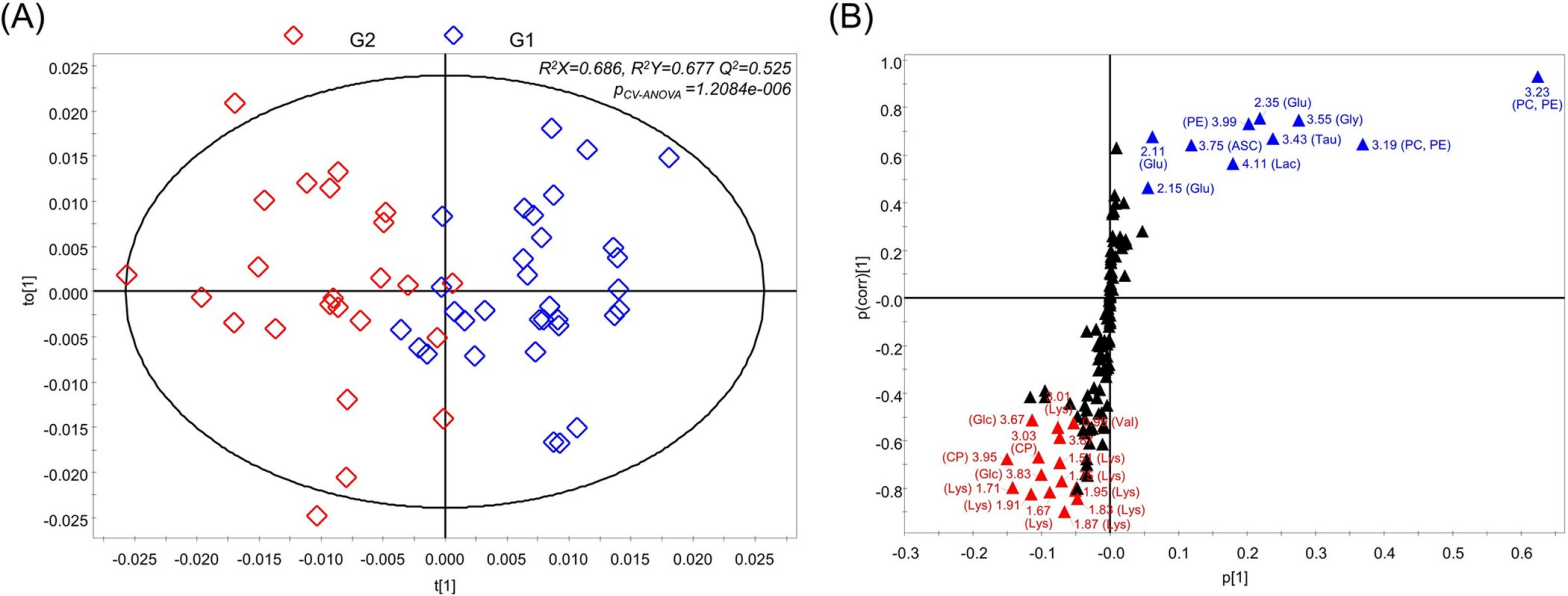
OPLS-DA score plot (A) and S-plot (B) of tonsil tissue. (◇, G1; ◇, G2) (ASC, Ascorbate; CP, Creatine phosphate; Glc, Glucose; Glu, Glutamate; Lac, Lactate; Lys, Lysine; PC, Phosphocholine; PE, Phosphoethanolamine; Tau, Taurine; Val, Valine).

For effective comparison of differences by age, OPLS-DA was performed with two age groups. The quality of the model was evaluated using parameters, R^2^, which corresponds to the goodness of fit, Q^2^, which indicates the goodness of prediction, and the *p* value obtained from analysis of variance testing of cross-validated predictive residuals (CV-ANOVA) for assessing the reliability (*p*_CV-ANOVA_, S2 Table). In the comparison of G1 and G2 independently of gender, the OPLS-DA score plot shows a clear separation with good validation parameters (R^2^X = 0.686, R^2^Y = 0.677, Q^2^ = 0.525, and *p*_CV-ANOVA_ < 0.05, Fig 3A). In addition, OPLS-DA model shows 91.07% of correct classification (S3 Table). The S-plot of OPLS-DA presented the components that strongly contributed to the group separation (Fig 3B). Variables with high model influence (|p| > 0.05) and reliability (|p(corr)| > 0.5) were identified as discriminating variables.

### Statistical analysis reveals metabolites associated with age and gender

**Fig 4:**
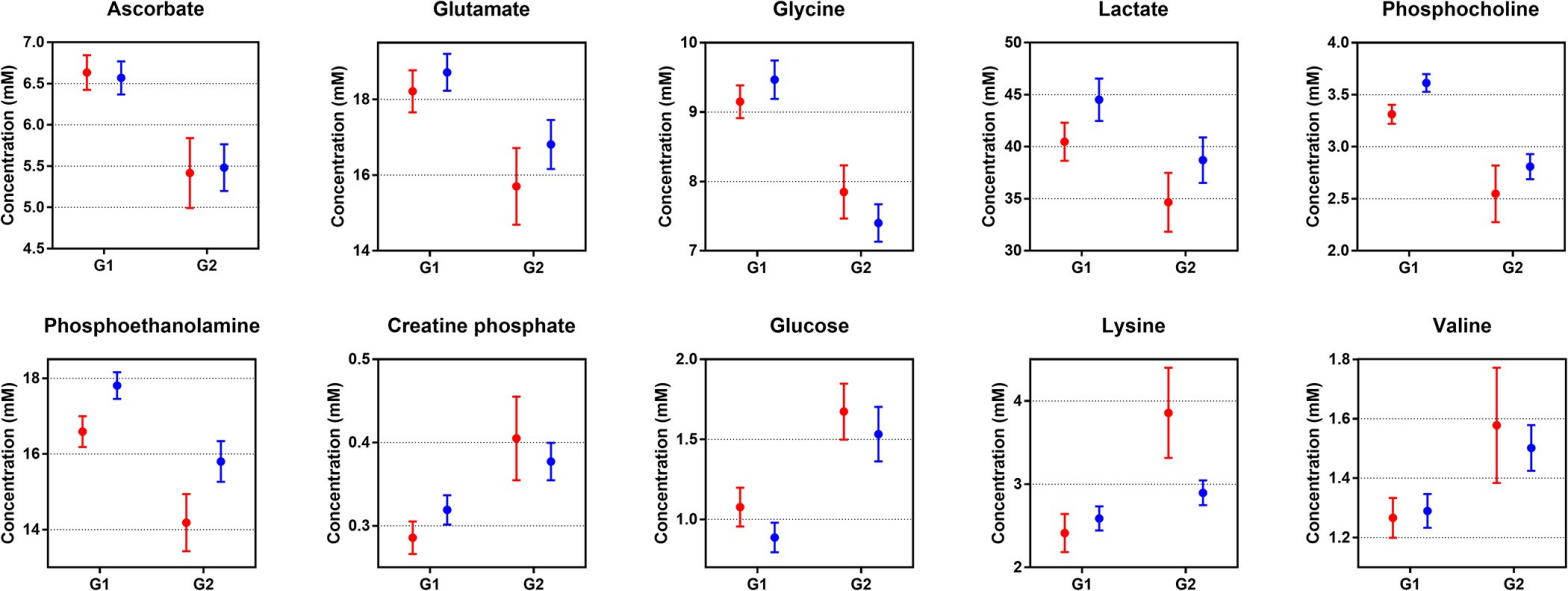
The concentration of metabolites in tonsil tissue according to age and gender. The levels of metabolites were significantly different depending on age. (red, female; blue, male).

Two-way ANOVA revealed whether the age and gender significantly influence on the quantified metabolic profiles (Table 2 and Fig 4). The levels of 10 metabolites were significantly different depending on age (FDR-adjusted *p* < 0.05). Except formate, all metabolites highlighted in the S-plot. Phosphocholine, phosphoethanolamine, and formate were influenced by gender (*p* < 0.05). However, the gender-based effects were no longer significant after adjusting for FDR (FDR-adjusted *p >* 0.05). There was no significant interaction effect between age and gender (FDR-adjusted *p >* 0.05). Because gender has an impact on metabolic profiles, partial correlation analysis was performed to control for the effect of gender to determine which metabolites are directly correlated with age (Table 3). After adjusting for gender, age showed a statistically significant linear relationship. There was a strong negative partial correlation in phosphocholine, glycine, ascorbate, and phosphoethanolamine levels with age and a strong positive correlation in glucose levels (│*r*│> 0.5, FDR-adjusted *p* < 0.05).

**Table 2:** Two-way ANOVA results on the influence of age and gender on the metabolites of tonsil tissue (FDR, FDR-adjusted *p*).

Factor	Gender			Age			Interaction			
Metabolites	F	*p*	FDR	F	*p*	FDR	F	*p*	FDR
**Phosphocholine**	5.152	0.027	0.216	40.958	4.E-08	2.E-06	0.023	0.881	0.933
**Glycine**	0.019	0.890	0.949	37.721	1.E-07	2.E-06	1.482	0.229	0.538
**Ascorbate**	0.004	0.949	0.949	17.241	1.E-04	9.E-04	0.050	0.823	0.933
**Glucose**	1.267	0.266	0.773	17.644	1.E-04	9.E-04	0.024	0.877	0.933
**Phosphoethanolamine**	6.968	0.011	0.198	17.907	9.E-05	9.E-04	0.137	0.713	0.901
**Creatine phosphate**	0.208	0.650	0.949	10.537	2.E-03	0.010	1.422	0.238	0.538
**Glutamate**	1.164	0.286	0.773	10.380	2.E-03	0.010	0.189	0.665	0.887
**Lysine**	1.101	0.299	0.773	9.726	3.E-03	0.014	5.978	0.018	0.342
**Valine**	0.021	0.884	0.949	8.523	5.E-03	0.020	0.311	0.579	0.881
**Lactate**	2.996	0.089	0.534	6.469	0.014	0.049	1.E-05	0.997	0.997

**Table 3:** Pearson partial correlation coefficients between age and metabolite concentrations by adjusted gender (FDR, FDR-adjusted *p*).

Metabolites	Correlation coefficient (*r*)	*p*	FDR
**Phosphocholine**	-0.692	5.E-09	2.E-07
**Glycine**	-0.653	6.E-08	1.E-06
**Glucose**	0.614	6.E-07	8.E-06
**Ascorbate**	-0.539	2.E-05	2.E-04
**Phosphoethanolamine**	-0.529	3.E-05	2.E-04
**Glutamate**	-0.461	4.E-04	0.002
**Creatine phosphate**	0.425	0.001	0.006
**Lysine**	0.398	0.003	0.011
**Valine**	0.383	0.004	0.014

To validate the findings, we quantified metabolites that significantly influenced by age in intact tissue in the spectra of tissue extraction and conducted the same analyses. The trends in metabolites of tissue extraction show similar age-related patterns, consistent with results observed in intact tissues (S3 Table). Although only phosphocholine, glycine, and creatine phosphate remained significant after FDR correction (FDR-adjusted *p* < 0.05, S4 Table), statistically relevant changes (*p* value < 0.05) were found for glutamate, glucose, and phosphoethanolamine. Furthermore, these metabolites were demonstrated significant correlations with age (FDR-adjusted p < 0.05, S5 Table).

## Discussion

PT is a B cell-predominant lymphoid organ that is easily accessible through the oral cavity because it is located in the pharynx and exposed. Unlike removal of other lymphoid organs, which is mainly owing to cancer, most tonsillectomies are performed because of chronic tonsillitis or obstructive sleep apnea caused by chronic tonsillar hypertrophy [7, 15, 16]. In addition, because the immunological function of the PT rapidly decreases after adolescence, it is optimal to study changes in lymphoid organs according to age [7, 8, 10]. There has been debate over the usefulness of tonsillectomy for several decades, but its frequency accounts for the largest number of otolaryngology surgeries; therefore, obtaining tissue samples is easy [15, 16].

In the present study, we found that phosphocholine, glycine, ascorbate, glucose, phosphoethanolamine, creatine phosphate, glutamate, lysine, valine, and, lactate levels were distinct between pediatric and adult groups. In the partial correlation analysis, glucose levels increased with age, and glycine, phosphocholine, phosphoethanolamine, and ascorbate levels decreased with age. These metabolites showed similar trends in the results of extract solutions obtained from the same PT samples. Most metabolites were significantly affected by age, although the statistical significance was slightly lower compared to the results obtained from intact PT. Based on these findings, we propose these metabolites as potential metabolic signatures for aging in PT.

Because lymphocytes have different energy demands according to the differentiation process, the metabolic profile of the PT may vary depending on which cell is predominant [17, 18]. Naive B and T cells require minimal energy production, and B cells require less energy than T cells [19, 20]. Resting B cells are more dependent on OXPHOS, and T cells are more dependent on aerobic glycolysis [19, 20].

When T and B cells are activated by exposure to antigens, large amounts of energy are required. The synthesis of lipid membranes, nucleic acids, and proteins takes precedence in T cells [21, 22]. Glycolytic activity increases, leading to a large uptake of glucose through Glut1. In particular, aerobic glycolysis is essential, and some glucose enters the TCA cycle [20, 23]. In addition, glutaminolysis is increased, and glutamine is converted to α-ketoglutarate, which is fuel for the TCA cycle [24]. Activated B cells rapidly increase energy production through the PI3K pathway, and increase oxygen consumption and glutamine uptake to meet energy demands through the TCA cycle and OXPHOS [25–27]. After the germinal center reaction, differentiated memory B cells survive with very little energy for long periods, and OXPHOS is used for energy production [20, 28].

With age, the number of memory B cells increases, immunologic activity decreases, and glycolytic activity decreases [10, 20]. In our study, lower glucose levels with pediatrics were the same as the previously reported low glucose levels in some cancer tissues [29–31]. Although glucose uptake increases through upregulation of Glut1, it is likely that depletion of glucose was observed owing to a large increase in glycolytic activity for cell proliferation. Similarly, increased glutamate levels in the pediatric group reflect increased glutaminolysis, and increased glycine levels reflect enhanced nucleotide synthesis through glycolysis and the serine-glycine biosynthesis pathways [31–34].

Phosphocholine and phosphoethanolamine are intermediates in the biosynthesis pathway of phosphatidylcholine and phosphatidylethanolamine, respectively, which are major lipids in cellular membranes [35, 36]. Previous studies have observed that levels of these phospholipids in plasma decrease with age, suggesting that their metabolism is closely related to the aging process [37, 38]. In this study, phosphocholine and phosphoethanolamine levels were strongly negatively correlated with age. Phospholipid metabolism is upregulated during B cell differentiation during germinal center reaction [39]. In this process, choline kinase and ethanolamine kinase are overexpressed and their activity is increased, and choline and ethanolamine are converted into phosphocholine and phosphoethanolamine, respectively [39–41]. Thus, higher levels of phosphocholine and phophoethanolamine in pediatric group than adult are consistent with increased membrane turnover reflecting cell proliferation [29].

However, it is not clear why ascorbate levels increased in the pediatric group. Ascorbate is an essential nutrient that cannot be synthesized naturally in the human body and must be supplied through an extrinsic source [42, 43]. Ascorbate enhances phagocytosis and chemotaxis of phagocytes in the immune system, but its role in lymphocytes is not distinct [44]. However, it has been reported that the intracellular ascorbate concentration of lymphocytes is up to 80-fold higher than that of plasma, suggesting a specific role of ascorbate in these cells [45]. Ascorbate promotes maturation of T cells, and it could be toxic to T cells at excessive levels [43]. However, the role of ascorbate in B cells is less clear and controversial. One in vitro study on mouse spleen B cells showed that ascorbate increases the production of IgM in a dose-dependent manner, whereas another study showed a dose-dependent increase in apoptosis [46, 47]. In a study on human peripheral blood lymphocytes in vitro, the number of IgM- and IgG-secreting cells increase in ascorbate-treated cultures [48]. Although the role of ascorbate in B cells is not clear, it is presumed, considering its role in T cells, that increased ascorbate levels in the pediatric group are the result of increased uptake through sodium-dependent vitamin C transporters [49].

One point to mention is the unusually high lactate levels found in both pediatric and adult subjects. Physiological lactate concentration levels in blood and tissues usually range around 1.5 to 3 mM. But in conditions like rheumatoid arthritis and cancer, they can surge up to 30 to 40 mM [50]. During the inflammatory response, activated T lymphocytes trigger a metabolic shift towards aerobic glycolysis to meet their energy needs, which in turn increases lactate. Several studies support this finding, showing a rise in intracellular lactate concentration, increased expression of glucose and lactate transporters, higher glucose uptake, and more glycolytic enzymes and LDH in activated T lymphocytes [51]. In this study, we measured lactate concentrations to be about 35 to 45 mM in both children and adults. This level might represent the physiological norm for the PT, given that they are constantly exposed to airborne and alimentary pathogens and that our subjects had no episodes of tonsillitis for six months. Still, the lack of studies reporting on lactate concentrations in lymph nodes and tonsil tissues signals a need for further research.

Our study has two limitations. First, the adolescent group (ages 11 to 17) was excluded. Since we had difficulty in obtaining their samples and intended to focus on the clear differences between the pediatric and adult groups. It would have been ideal to have the adolescent group included as it would have allowed us to draw a natural and clear direction of the metabolic change based on age. However, previous immunological studies have shown that immune function decreases linearly as age increases from pediatric to adolescent to adult [6, 10]. So, it is estimated that the changes in the metabolic profile of the tonsils in the adolescent group would also be between the pediatric and adult groups. Second, we did not consider the body weight and size of the PT. The size of the PT is larger in obese children than in normal-weight children with sleep-disordered breathing [52]. There is currently no research that suggests body weight impacts the immune system of the PT. This study compared the immune function of the PT between pediatrics and adults, which have apparent immunological differences. Thus, it is estimated that body weight will have a minimal effect on the study results. However, with the recurrence of tonsillitis, the size of the PT increases due to follicular hyperplasia, so future studies will aim to secure a sufficient number of samples and consider factors such as the size of the tonsils and the patient’s weight [53].

The lack of prior metabolomic studies related to aging the PT or lymph nodes makes the clinical interpretation of this research difficult. From a perspective of established glucose metabolism, the decrease in glucose concentration and increase in lactate in children suggests active aerobic glycolysis to satisfy high cellular energy demand. This process is similar to what occurs in inflammatory situations and cancer cells [29–31, 50, 51]. Although previous studies reported at histological and immunological changes with age, this research solidifies the understanding of decreasing immunometabolic activity in the PT [7–10].

However, for more clinically significant judgments, further investigation is needed in two areas. First, comparing the pace of immune metabolic changes in the PT due to aging with shifts in other lymphoid organs could prove intriguing. Secondly, gathering a larger, more evenly age-distributed set of samples might reveal the age at which the immune metabolism in the PT significantly declines. These potential findings could provide beneficial perspectives on the optimal age for tonsillectomy and its justification.

To the best of our knowledge, this is the first study to use HR-MAS NMR to analyze PT tissues. Most clinical studies of metabolomics have mainly focused on the identification of disease metabolites. Among them, there are few studies on metabolites in head and neck cancer, and there are no studies that analyze metabolites limited to tonsil cancer [54, 55]. In this respect, this study also provides valuable basic data for the study of tonsil cancer.

## Conclusion

In conclusion, phosphocholine, glycine, ascorbate, glucose, phosphoethanolamine, creatine phosphate, glutamate, lysine, valine, and, lactate were distinct metabolites in the pediatric and adult groups. Glucose levels increased with age, and glycine, phosphocholine, phosphoethanolamine, and ascorbate levels tended to decrease with age. We confirmed the decrease in immunometabolic activity in adults through metabolomic analysis, which had been anticipated from previous histological and immunological studies on the PT. Also, it is expected that this study will improve our understanding of metabolic changes in the PT with aging and will serve as a basis for future tonsil-related metabolomic studies.

## Supporting information

S1 Dataset (XLSX)

S1 Fig Hotelling’s T^2^ plots of the PCA models for outlier detection. (DOCX)

S1 Table Identified and quantified metabolites in tonsil tissue from ^1^H HR-MAS NMR spectra. (DOCX)

S2 Table The output of the CV-ANOVA of OPLS-DA model. (DOCX)

S3 Table Identified and quantified metabolites in extract solutions of PT from ^1^H NMR spectra. (DOCX)

S4 Table Two-way ANOVA results on the influence of age and gender on the metabolites in extract solutions of PT. (DOCX)

S5 Table Pearson partial correlation coefficients between age and metabolite concentrations in extract solutions by adjusted gender. (DOCX)
