# Supplementary figures and images for: Differences in the metabolomic profile of the human palatine tonsil between pediatrics and adults
Source: PLoS One. 2023 Jul 31;18(7):e0288871. doi: 10.1371/journal.pone.0288871 (PMC10389742; doi:10.1371/journal.pone.0288871)

**S1 Fig. Hotelling's T2 plots of the PCA models for outlier detection.**


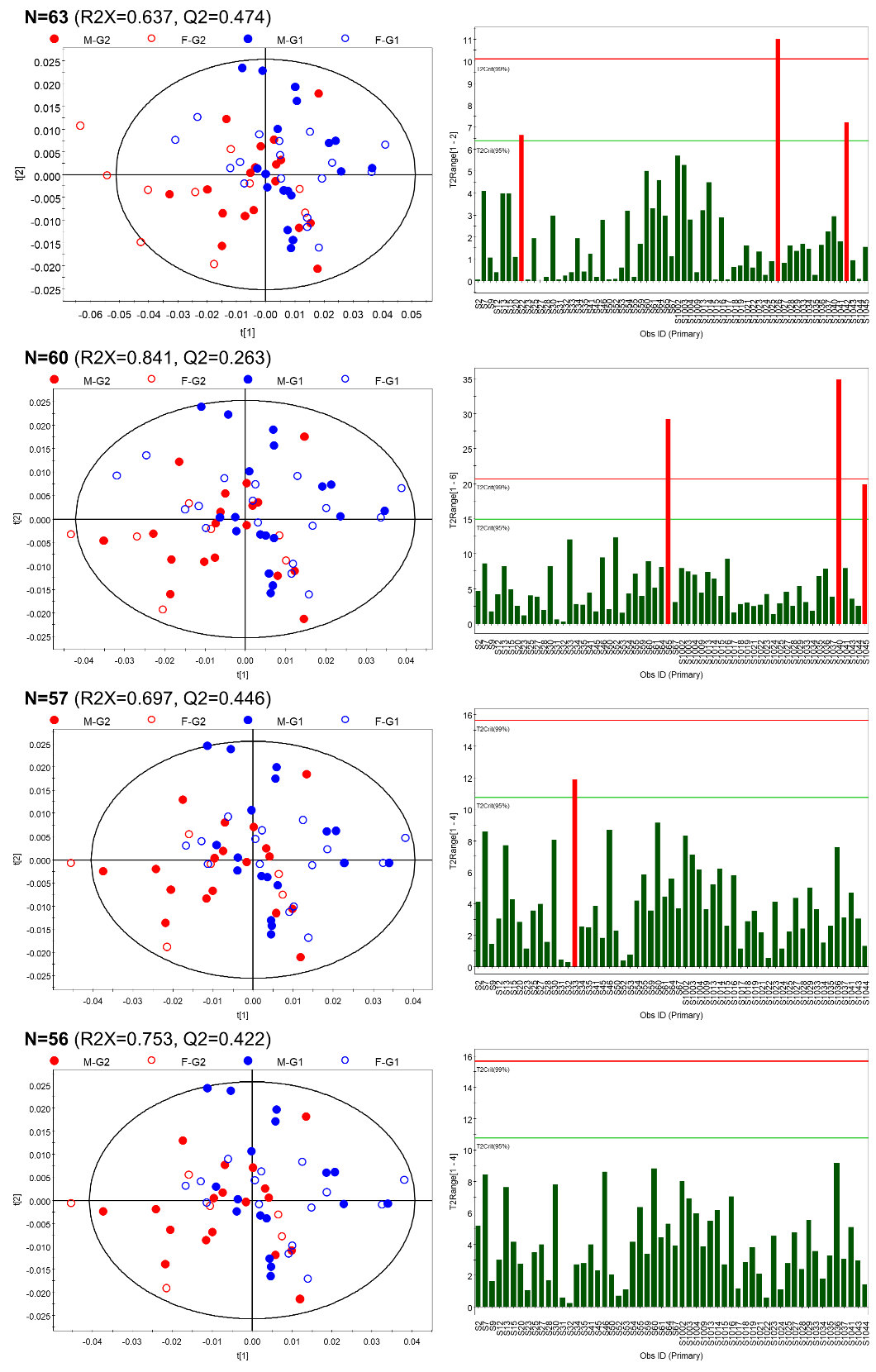

Supplement: S1 Fig — (DOCX) [file pone.0288871.s002.docx]
