# Supplementary material for: Differences in the metabolomic profile of the human palatine tonsil between pediatrics and adults
Source: PLoS One. 2023 Jul 31;18(7):e0288871. doi: 10.1371/journal.pone.0288871 (PMC10389742; doi:10.1371/journal.pone.0288871)
Supplement: S1 Table — (DOCX) [file pone.0288871.s003.docx]

**S1 Table. Identified and quantified metabolites in tonsil tissue from ^1^H HR-MAS NMR spectra.**

| **Metabolites (mM)** | **G1 (n=32)** | | **G2 (n=24)** | | **M-G1 (n=17)** | | **M-G2 (n=18)** | | **F-G1 (n=15)** | | **F-G2 (n=6)** | |
| --- | --- | --- | --- | --- | --- | --- | --- | --- | --- | --- | --- | --- |
|  | **Mean** | **SD** | **Mean** | **SD** | **Mean** | **SD** | **Mean** | **SD** | **Mean** | **SD** | **Mean** | **SD** |
| **Acetate** | 0.602 | 0.05 | 0.896 | 0.145 | 0.597 | 0.086 | 0.983 | 0.189 | 0.608 | 0.048 | 0.633 | 0.072 |
| **Alanine** | 7.026 | 0.184 | 7.564 | 0.164 | 7.078 | 0.268 | 7.566 | 0.192 | 6.967 | 0.259 | 7.558 | 0.344 |
| **Ascorbate** | 6.598 | 0.143 | 5.465 | 0.233 | 6.567 | 0.201 | 5.481 | 0.283 | 6.632 | 0.21 | 5.416 | 0.423 |
| **Asparagine** | 1.001 | 0.04 | 0.947 | 0.044 | 1.035 | 0.048 | 0.92 | 0.042 | 0.963 | 0.066 | 1.029 | 0.124 |
| **Aspartate** | 4.388 | 0.117 | 4.314 | 0.219 | 4.429 | 0.13 | 4.179 | 0.278 | 4.342 | 0.206 | 4.717 | 0.228 |
| **Choline** | 2.515 | 0.081 | 2.392 | 0.101 | 2.547 | 0.09 | 2.373 | 0.118 | 2.479 | 0.142 | 2.447 | 0.21 |
| **Creatine** | 1.338 | 0.09 | 1.607 | 0.088 | 1.278 | 0.099 | 1.634 | 0.108 | 1.407 | 0.157 | 1.525 | 0.148 |
| **Creatine phosphate** | 0.303 | 0.013 | 0.384 | 0.021 | 0.319 | 0.017 | 0.377 | 0.022 | 0.286 | 0.02 | 0.405 | 0.05 |
| **Ethanolamine** | 0.982 | 0.057 | 1.074 | 0.058 | 0.913 | 0.056 | 1.051 | 0.069 | 1.061 | 0.101 | 1.142 | 0.109 |
| **Formate** | 0.689 | 0.054 | 0.484 | 0.032 | 0.59 | 0.066 | 0.47 | 0.035 | 0.801 | 0.081 | 0.525 | 0.079 |
| **Fumarate** | 0.379 | 0.012 | 0.347 | 0.018 | 0.39 | 0.02 | 0.371 | 0.018 | 0.366 | 0.013 | 0.273 | 0.035 |
| **Glucose** | 0.976 | 0.076 | 1.568 | 0.134 | 0.887 | 0.092 | 1.533 | 0.171 | 1.077 | 0.121 | 1.673 | 0.175 |
| **Glutamate** | 18.482 | 0.365 | 16.534 | 0.546 | 18.718 | 0.489 | 16.811 | 0.648 | 18.215 | 0.557 | 15.703 | 1.012 |
| **Glutamine** | 2.145 | 0.147 | 2.722 | 0.223 | 1.918 | 0.214 | 2.745 | 0.263 | 2.401 | 0.183 | 2.652 | 0.45 |
| **Glutathione** | 2.222 | 0.08 | 2.502 | 0.122 | 2.283 | 0.078 | 2.568 | 0.134 | 2.154 | 0.149 | 2.303 | 0.284 |
| **Glycerol** | 1.049 | 0.055 | 1.036 | 0.081 | 1.054 | 0.079 | 0.938 | 0.073 | 1.043 | 0.079 | 1.33 | 0.211 |
| **Glycine** | 9.319 | 0.184 | 7.512 | 0.223 | 9.468 | 0.277 | 7.4 | 0.269 | 9.15 | 0.236 | 7.848 | 0.383 |
| **Inosine** | 0.425 | 0.033 | 0.324 | 0.031 | 0.398 | 0.039 | 0.339 | 0.038 | 0.457 | 0.055 | 0.277 | 0.053 |
| **Isoleucine** | 0.396 | 0.025 | 0.507 | 0.049 | 0.407 | 0.035 | 0.474 | 0.05 | 0.383 | 0.037 | 0.604 | 0.129 |
| **Lactate** | 42.617 | 1.41 | 37.69 | 1.791 | 44.51 | 2.042 | 38.703 | 2.182 | 40.473 | 1.835 | 34.648 | 2.823 |
| **Leucine** | 1.47 | 0.059 | 1.636 | 0.079 | 1.506 | 0.089 | 1.575 | 0.076 | 1.429 | 0.079 | 1.819 | 0.213 |
| **Lysine** | 2.506 | 0.131 | 3.136 | 0.189 | 2.589 | 0.144 | 2.896 | 0.149 | 2.413 | 0.229 | 3.856 | 0.542 |
| **Methionine** | 0.508 | 0.023 | 0.564 | 0.029 | 0.49 | 0.031 | 0.573 | 0.031 | 0.529 | 0.035 | 0.538 | 0.073 |
| **myo-Inositol** | 4.973 | 0.145 | 5.119 | 0.205 | 5.018 | 0.208 | 5.255 | 0.238 | 4.923 | 0.207 | 4.712 | 0.389 |
| **Niacinamide** | 0.5 | 0.024 | 0.477 | 0.024 | 0.523 | 0.035 | 0.488 | 0.028 | 0.475 | 0.031 | 0.444 | 0.047 |
| **Oxypurinol** | 16.016 | 0.982 | 19.045 | 1.816 | 14.983 | 1.093 | 20.519 | 2.29 | 17.187 | 1.681 | 14.62 | 1.391 |
| **Phenylalanine** | 0.591 | 0.032 | 0.682 | 0.043 | 0.621 | 0.044 | 0.636 | 0.034 | 0.559 | 0.046 | 0.819 | 0.132 |
| **Phosphocholine** | 3.472 | 0.067 | 2.744 | 0.112 | 3.613 | 0.084 | 2.809 | 0.12 | 3.312 | 0.091 | 2.548 | 0.272 |
| **Phosphoethanolamine** | 17.237 | 0.284 | 15.396 | 0.46 | 17.809 | 0.352 | 15.8 | 0.535 | 16.588 | 0.406 | 14.181 | 0.754 |
| **Proline** | 2.251 | 0.096 | 2.443 | 0.143 | 2.165 | 0.111 | 2.231 | 0.145 | 2.35 | 0.162 | 3.08 | 0.231 |
| **sn-Glycero-3-phosphocholine** | 1.561 | 0.074 | 1.412 | 0.083 | 1.509 | 0.112 | 1.451 | 0.098 | 1.621 | 0.097 | 1.293 | 0.154 |
| **Taurine** | 25.138 | 0.537 | 25.49 | 0.918 | 25.306 | 0.689 | 25.884 | 1.041 | 24.947 | 0.863 | 24.311 | 2.019 |
| **Threonine** | 2.034 | 0.078 | 2.138 | 0.087 | 2.059 | 0.097 | 2.056 | 0.091 | 2.006 | 0.127 | 2.384 | 0.192 |
| **Tyrosine** | 0.759 | 0.033 | 0.86 | 0.048 | 0.809 | 0.046 | 0.817 | 0.038 | 0.704 | 0.043 | 0.988 | 0.153 |
| **Uridine** | 0.664 | 0.032 | 0.579 | 0.041 | 0.67 | 0.052 | 0.592 | 0.047 | 0.657 | 0.036 | 0.541 | 0.086 |
| **Valine** | 1.279 | 0.043 | 1.521 | 0.073 | 1.29 | 0.057 | 1.502 | 0.077 | 1.266 | 0.067 | 1.578 | 0.194 |
