# Supplementary material for: Differences in the metabolomic profile of the human palatine tonsil between pediatrics and adults
Source: PLoS One. 2023 Jul 31;18(7):e0288871. doi: 10.1371/journal.pone.0288871 (PMC10389742; doi:10.1371/journal.pone.0288871)
Supplement: S2 Table — (DOCX) [file pone.0288871.s004.docx]

**S2 Table. The output of the CV-ANOVA of OPLS-DA model.**

|  | **SS** | **DF** | **MS** | **F** | **p** | **SD** |
| --- | --- | --- | --- | --- | --- | --- |
| **Total corr.** | 55 | 55 | 1 |  |  | 1 |
| **Regression** | 28.8661 | 6 | 4.81101 | 9.02044 | 1.2084e-006 | 2.1934 |
| **Residual** | 26.1339 | 49 | 0.533346 |  |  | 0.730305 |
