# Supplementary material for: Differences in the metabolomic profile of the human palatine tonsil between pediatrics and adults
Source: PLoS One. 2023 Jul 31;18(7):e0288871. doi: 10.1371/journal.pone.0288871 (PMC10389742; doi:10.1371/journal.pone.0288871)
Supplement: S3 Table — (DOCX) [file pone.0288871.s005.docx]

**S3** **Table. Identified and quantified metabolites in extract solutions of PT from ^1^H NMR spectra**

| **Metabolites (mM)** | **δ** **^1^H (ppm) ^*^** | **G1 (n=32)** | | **G2 (n=24)** | | **M-G1 (n=17)** | | **M-G2 (n=18)** | | **F-G1 (n=15)** | | **F-G2 (n=6)** | | |
| --- | --- | --- | --- | --- | --- | --- | --- | --- | --- | --- | --- | --- | --- | --- |
|  |  | **Mean^**^** | **SD** | **Mean^**^** | **SD** | **Mean^**^** | **SD** | **Mean^**^** | **SD** | **Mean^**^** | **SD** | **Mean^**^** | **SD** | |
| **Ascorbate** |  | 0.172 | 0.0977 | 0.154 | 0.0704 | 0.199 | 0.0231 | 0.139 | 0.0166 | 0.140 | 0.0241 | 0.201 | 0.0200 |  |
| **Creatine phosphate** |  | 0.0066 | 0.0025 | 0.0105 | 0.0046 | 0.00640 | 0.00073 | 0.011 | 0.00107 | 0.00687 | 0.00051 | 0.00879 | 0.00182 |  |
| **Glucose** |  | 0.0206 | 0.0090 | 0.0406 | 0.0413 | 0.0185 | 0.0017 | 0.0367 | 0.0100 | 0.0230 | 0.0028 | 0.0524 | 0.0161 |  |
| **Glutamate** |  | 0.670 | 0.0751 | 0.609 | 0.109 | 0.683 | 0.0161 | 0.614 | 0.0260 | 0.654 | 0.0216 | 0.593 | 0.0462 |  |
| **Glycine** |  | 0.308 | 0.0440 | 0.229 | 0.0306 | 0.312 | 0.0100 | 0.232 | 0.0077 | 0.304 | 0.0124 | 0.222 | 0.0099 |  |
| **Lactate** |  | 1.583 | 0.243 | 1.459 | 0.237 | 1.591 | 0.0480 | 1.463 | 0.0541 | 1.573 | 0.0756 | 1.448 | 0.115 |  |
| **Lysine** |  | 0.0253 | 0.0080 | 0.0291 | 0.0068 | 0.0255 | 0.0018 | 0.0284 | 0.0017 | 0.0251 | 0.0023 | 0.0312 | 0.0022 |  |
| **Phosphocholine** |  | 0.110 | 0.0171 | 0.0829 | 0.0166 | 0.115 | 0.0029 | 0.0847 | 0.0041 | 0.1031 | 0.0051 | 0.0775 | 0.0058 |  |
| **Phosphoethanolamine** |  | 0.688 | 0.0854 | 0.631 | 0.120 | 0.716 | 0.0155 | 0.627 | 0.0295 | 0.657 | 0.0251 | 0.643 | 0.0457 |  |
| **Valine** |  | 0.0490 | 0.0072 | 0.0485 | 0.0076 | 0.0483 | 0.0016 | 0.0498 | 0.0019 | 0.0498 | 0.0021 | 0.0445 | 0.0015 |  |
| ^*^ All chemical shifts are based on the TSP-d_4_ calibrated at δ 0.0 ppm.  ^**^ Each metabolite concentration was calculated using 2 mM TSP-d_4_ as an internal standard. | | | | | | | | | | | | | |  |
