# Supplementary material for: Differences in the metabolomic profile of the human palatine tonsil between pediatrics and adults
Source: PLoS One. 2023 Jul 31;18(7):e0288871. doi: 10.1371/journal.pone.0288871 (PMC10389742; doi:10.1371/journal.pone.0288871)
Supplement: S4 Table — (DOCX) [file pone.0288871.s006.docx]

**S4** **Table. Two-way ANOVA results on the influence of age and gender on the metabolites in extract solutions of PT**

| **Factor** | **Gender** | | | **Age** | | | **Interaction** | | |
| --- | --- | --- | --- | --- | --- | --- | --- | --- | --- |
| **Metabolites** | **F** | ***p*** | **FDR** | **F** | ***p*** | **FDR** | **F** | ***p*** | **FDR** |
| Glycine | 0.57 | 0.454 | 0.871 | 54.474 | 1.22E-09 | 6.47E-08 | 0.021 | 0.884 | 0.937 |
| Phosphocholine | 5.028 | 0.029 | 0.769 | 40.579 | 4.99E-08 | 1.32E-06 | 0.277 | 0.601 | 0.816 |
| Creatine phosphate | 0.269 | 0.606 | 0.892 | 14.701 | 0.000342 | 0.006 | 1.722 | 0.195 | 0.627 |
| Glucose | 1.176 | 0.283 | 0.831 | 7.97 | 0.007 | 0.053 | 0.468 | 0.497 | 0.775 |
| Glutamate | 1.028 | 0.315 | 0.831 | 6.807 | 0.012 | 0.080 | 0.022 | 0.884 | 0.937 |
| Phosphoethanolamine | 1.252 | 0.268 | 0.831 | 5.352 | 0.025 | 0.147 | 1.632 | 0.207 | 0.627 |
| Lactate | 0.057 | 0.812 | 0.936 | 3.499 | 0.067 | 0.197 | 0 | 0.985 | 0.985 |
| Lysine | 0.144 | 0.706 | 0.922 | 3.499 | 0.067 | 0.197 | 0.491 | 0.487 | 0.775 |
| Ascorbate | 0.431 | 0.514 | 0.871 | 0.784 | 0.38 | 0.65 | 6.118 | 0.017 | 0.451 |
| Valine | 0.229 | 0.634 | 0.908 | 0.138 | 0.712 | 0.889 | 2.45 | 0.124 | 0.627 |
