# Supplementary material for: Differences in the metabolomic profile of the human palatine tonsil between pediatrics and adults
Source: PLoS One. 2023 Jul 31;18(7):e0288871. doi: 10.1371/journal.pone.0288871 (PMC10389742; doi:10.1371/journal.pone.0288871)
Supplement: S5 Table — (DOCX) [file pone.0288871.s007.docx]

**S5 Table. Pearson partial correlation coefficients between age and metabolite concentrations in extract solutions by adjusted gender**

| **Metabolites** | **correlation r** | ***p* value** | **FDR** |
| --- | --- | --- | --- |
| Glycine | -0.718 | 7.E-10 | 4.E-08 |
| Phosphocholine | -0.694 | 4.E-09 | 1.E-07 |
| Creatine phosphate | 0.604 | 1.E-06 | 2.E-05 |
| Glucose | 0.504 | 9.E-05 | 0.001 |
| Glutamate | -0.392 | 0.003 | 0.031 |
| Phosphoethanolamine | -0.383 | 0.004 | 0.031 |
| Lysine | 0.326 | 0.015 | 0.073 |
| Lactate | -0.266 | 0.049 | 0.188 |
| Ascorbate | -0.186 | 0.174 | 0.436 |
| Valine | -0.005 | 0.971 | 0.991 |
